# Janus kinase inhibitor, tofacitinib, in refractory juvenile dermatomyositis: a retrospective multi-central study in China

**DOI:** 10.1186/s13075-023-03170-z

**Published:** 2023-10-18

**Authors:** Junmei Zhang, Li Sun, XinWei Shi, Shipeng Li, Cuihua Liu, Xiaoqing Li, Meiping Lu, Jianghong Deng, Xiaohua Tan, Wanzhen Guan, Guomin Li, Xinran Wen, Ping Liu, Caifeng Li

**Affiliations:** 1 Department of Rheumatology, Beijing Children’s Hospital, Capital Medical University, National Center for Children’s Health, Beijing, 100045 China; 2 Department of Rheumatology, Children’s Hospital of Fudan University, National Children’s Medical Center, No. 399 Wanyuan Road, Shanghai, 201102 China; 3 Department of Nephrology and Rheumatology, Children’s Hospital Affiliated to Zhengzhou University, Zhengzhou, 450018 China; 4 Department of Nephrology and Rheumatology, Henan Children’s Hospital, Zhengzhou Children’s Hospital, Zhengzhou, 450018 China; 5 Department of Rheumatology and Immunology, Xi’an Children’s Hospital, Xi’an, 710002 China; 6 Department of Rheumatology Immunology and Allergy, Children’s Hospital of Zhejiang University School of Medicine, Hangzhou, 310003 China

**Keywords:** Juvenile dermatomyositis, Tofacitinib, Biologic therapy, Janus kinase inhibitor, Refractory

## Abstract

**Objectives:**

Juvenile dermatomyositis (JDM) is a chronic autoimmune disease. Some patients remain in an active state even though they were administrated with a combination of corticosteroid and methotrexate. Existing research has suggested that interferon and Janus kinase played an important role in pathogenesis. Existing research has suggested the efficacy of JAK inhibitors (JAKi). Our retrospective study aimed to investigate the efficacy of tofacitinib in refractory JDM patients.

**Methods:**

A total of eighty-eight patients in China who had been diagnosed with JDM and subjected to tofacitinib therapy for over 3 months were retrospectively analyzed. Skin and muscle manifestations were assessed using the Cutaneous Assessment Tool-binary method (CAT-BM), Childhood Myositis Assessment Scale (CMAS), and kinase. Pulmonary function was assessed using a high-resolution CT (computerized tomography) scan and pulmonary symptoms. All patients were subjected to regular follow-up, and core measures were assessed every 3 months after initiation. Furthermore, the data were analyzed using the Wilcoxon single test, Mann–Whitney *U* test, and chi-square test.

**Results:**

Compared with the baseline data, skin and muscle manifestations were found significantly improved during the respective follow-up visit. At the most recent follow-up, nearly 50% of patients achieved a clinical complete response and six patients received tofacitinib monotherapy. Sixty percent of patients suffering from interstitial lung disease well recovered on high-resolution CT. Seventy-five percent of patients showed a reduction in the size or number of calcinosis, and 25% of patients showed completely resolved calcinosis.

**Conclusion:**

In this study, the result suggested that tofacitinib therapy exerted a certain effect on skin manifestations, muscle manifestations, interstitial lung disease (ILD), calcinosis, as well as downgrade of medication. In-depth research should be conducted to focus on the correlation between the pathogenesis of JDM and JAKi.

## Introduction

Juvenile dermatomyositis (JDM) is a chronic autoimmune disease. It has been confirmed as the most prevalent subgroup of juvenile idiopathic inflammatory myopathies (IIM) [1]. JDM primarily affects skin and muscle and other organs can be affected. Characteristic cutaneous lesions are manifested as heliotrope rash and Gottron’s papules. Proximal muscles are primarily affected and then progressed to be symmetrical. Patients were diagnosed in accordance with Bohan and Peter’s criteria in 1975. Five criteria have been followed to define JDM, comprising symmetrical muscle weakness, abnormal muscle biopsy, elevation of kinase, abnormal electromyogram, and dermatological features [2]. Cutaneous manifestation is the main clinical expression of amyopathic JDM with minimal or absence of muscular involvement. JDM autoantibodies can fall into two subgroups: myositis-specific antibodies (MSAs) and myositis-associated antibodies (MAAs). Recent research has suggested that autoantibodies are associated with phenotype, treatment, as well as prognosis. For example, anti-MDA5 autoantibody is associated with rapidly progressive interstitial lung disease (RP-ILD) [3]. Macrophage activation syndrome (MAS) is a rare and fatal complication of JDM, whereas its occurrence has been underestimated. MAS is hard to treat and be properly assessed [4].

In accordance with treatment recommendations published in 2017 by EULAR, high doses of steroids combined with methotrexate (MTX) were recommended as the initial treatment of JDM. Intravenous immunoglobulin (IVIG) was useful in treating cutaneous manifestations. Mycophenolate mofetil (MMF) and cyclosporin A (CSA) were used in methotrexate-intolerant patients. Intravenous cyclophosphamide and biologics could be considered in treating patients with refractory JDM [5]. Although combined treatment yielded remission, nearly 40% of patients were still in active states [6]. These patients were hard to treat due to disease recurrence, failure of two or more disease-modifying antirheumatic drugs (DMARDs) therapy, and difficulty in taping the dosage of prednisolone.

Recently, there has been an emerging interest of interferon (IFN) in the pathogenesis of JDM. Elevated interferon levels in serum, muscle, and skin and increased expression of interferon-regulated gene (IRG) in JDM patients indicate the important role of interferon [7]. Interferon binds to its receptor and then activates IRG expression while producing cytokines via the Janus kinase (JAK)/Signal Transducers and Activators of Transcription (STAT) pathway [8]. JAK inhibitors (JAKi) blocking the JAK-STAT pathway had yielded satisfactory responses in treating interferonopathies with remission of symptoms and decreased IFN markers [9]. There were cases reported that JAK inhibitors had successfully treated refractory JDM and 50% of patients achieved clinical inactive disease (CID) after 6 months of initiation. However, their sample size was small (*n* = 10) [10]. Herein, 88 refractory JDM patients were reported in a multi-center in China treated with tofacitinib. The study aimed to evaluate the safety and efficacy of tofacitinib in treating refractory JDM patients.

## Methods

### Study population

This study was a retrospective study from May 1 to December 31 in the year of 2021. All patients were diagnosed based on Bohan and Peter’s criteria in 1975 [2]. Beijing Children’s Hospital, Children’s Hospital of Fudan University, Henan Children’s Hospital, Xi’an Children’s Hospital, and Children’s Hospital of Zhejiang University School of Medicine participated in this study. Patients were included if they were younger than 18, had ever received over 3 months of tofacitinib treatment, and regularly followed up in the respective center. Patients were excluded if receiving less than 3 months of tofacitinib treatment. Indications of starting tofacitinib therapy were active skin and muscle involvement.

### Ethical statement

This study gained approval from the Ethics Committee of a hospital on 1 Aug 2022 ([2022]-E-156-R). Written informed consent was obtained from the patient and their legal guardians.

### Data collection

All clinical information including clinical course and laboratory results were collected. Laboratory tests like liver and renal function, coagulation profile, and routine blood and urine tests were done during each follow-up. Myositis-specific antibodies (MSAs) and myositis-associated antibodies (MAAs) of five centers were evaluated by enzyme-linked immunosorbent assay (ELISA). Physical examination and essential images were done to assess disease activity. Cutaneous Assessment Tool-binary method (CAT-BM, skin disease activity score range 0–17, corresponding to “inactive,” “mild,” “moderate,” “severe,” and “very severe” were 0.7 ± 0.6, 5.0 ± 2.4, 8.0 ± 2.0, 9.8 ± 1.8, 10.5 ± 0.7; skin disease damage score range 0–11, corresponding to “mild,” “moderate,” and “severe” were 2.3 ± 1.6, 4.2 ± 2.0, 5.7 ± 1.7) and Childhood Myositis Assessment Scale (CMAS, score range 0–52, with high score indicate minimal disease) were used to evaluate skin involvement and muscle strength. Aspartate aminotransferase (AST, normal range 14–44 U/L), alanine aminotransferase (ALT, normal range 7–30 U/L), creatine kinase (CK, normal range 25–200 U/L), lactate dehydrogenase (LDH, normal range 110–295 U/L) were covered. Pulmonary function was investigated in accordance with respiratory symptoms and a high-resolution CT scan. International Myositis Assessment and Clinical Studies Group (IMACS) defined complete clinical response as more than 6 months of inactive disease (e.g., normal muscle strength, no skin rashes, and normal kinase under myositis treatment). Complete clinical remission was defined as over 6 months of inactive disease without treatment. Medical escalation was defined as an increase of over 25% of the original dose and as an increase of therapy due to increased disease activity [10]. Opportunistic infections (OI), gastrointestinal perforation, thromboembolism, malignancy, and thrombocytopenia were common adverse effects of tofacitinib and monitored carefully during the respective follow-up. Baseline (month = 0) was the time that tofacitinib was initiated.

### Statistical analysis

All data were studied using SPSS 20.0. The follow-up data were compared with the baseline data through the Wilcoxon single rank test and Mann–Whitney *U* test. The treatment response of MSAs and MAAs was compared using the chi-square test. *P* value < 0.05 indicated a difference with statistical significance.

## Results

### General information

Of all cases assessed, 49 (55.6%) were female and all Han Chinese. Median age of disease onset was 82.0 ± 4.1 months (ranging from 17 to 176 months). Table 1 lists the general information. At the time of the first prescription, 86 (97.7%) of patients had skin involvement. Their CAT activity score ranged from 0 to 6, with 33 inactive (38.3%), 51 (59.3%) mild and 2 (2.3%) moderate. CAT damage score ranged from 0 to 7, with 38 (43.2%) mild, 39 (44.3%) moderate, and 9 (10.2%) severe. The muscular system was the second most affected system with 65(73.9%) patients suffering from decreased muscle strength. Six (6.8%) patients had dysphagia. Medium CMAS at first prescription was 34.0 with 5 (5.9%) patients in severe states (less than 15 score). At first prescription, 17 (19.3%) patients suffered from calcinosis. Seven (8.0%) patients had lipoatrophy. A total of 50 (56.8%) patients were reported with interstitial lung disease (ILD) at first prescription, as indicated by the assessment result of high-resolution pulmonary CT scan and respiratory symptoms. The joint, digestive, and central nervous systems are also affected. All patients had finished myositis-specific antibodies (MSAs) and myositis-associated antibodies (MAAs) tests. Forty-seven patients (53.4%) had positive MSAs and MAAs. To be specific, anti-NXP2 and anti-MDA5 were most abundant. Seventeen out of 19 (89.4%) patients with positive anti-MDA antibodies presented with ILD at first prescription. Six patients (50.0%) with positive anti-NXP2 antibodies presented with ILD.

**Table 1:** General information at the first prescription of refractory JDM patients

Feature	Number (%) or median [IQR]
Sex	
Male	39 (44.3%)
Female	49 (55.7%)
Ethnicity	
Han Chinese	100%
Median age at disease onset	82.0 ± 4.1 months
Manifestation at disease onset	
Skin involvement	86 (97.9%)
Medium CAT activity score	2.7 ± 0.1
Medium CAT damage score	2.7 ± 0.2
Muscle involvement	65 (73.8%)
Medium CMAS	34.0 ± 1.2
Pulmonary involvement (ILD)	50 (56.8%)
Joint involvement	33 (37.5%)
Digestive system involvement	3 (3.4%)
Central nervous system involvement	5 (5.6%)
Calcinosis	17 (19.3%)
Myositis specific and associated antibodies	
Anti-TIF1γ	5 (5.6%)
Anti-NXP2	12 (13.6%)
Anti-MDA5	19 (21.5%)
Anti-Mi2	1 (1.1%)
Anti-SNP	1 (1.1%)
Anti-PL-7	1 (1.1%)
Anti-SSA	5 (5.6%)
Anti-Ku	1 (1.1%)
Anti-Jo	1 (1.1%)
Other antibodies (anti-SNP)	1 (1.1%)
Time at JAKi start	22.0 ± 2.7 months
Duration of JAKi therapy	10.1 ± 0.6 months

Indication of starting JAK inhibitor treatment comprised active skin and muscle involvement and failure with previous DMARDs and biologics. The median disease duration of starting of JAK inhibitor treatment and median time of JAK inhibitor treatment were 22.0 ± 2.7 months and 10.1 ± 0.6 months (ranging from 3 to 21 months). Fifteen (17.0%) patients were administrated with the combination of tofacitinib, steroids, and immunosuppressive agents at first prescription due to long medical history in external hospitals and high disease activity. The longest time from the first prescription to starting tofacitinib reached 141 months.

### Medication

As for previous medication, all cases used either prednisolone, immunosuppressant, or biologics as therapy. A combination of prednisolone (2 mg/kg) and methotrexate was used as first-line treatment in 50 (56.8%) patients. Fifty-one (57.9%) patients received methylprednisolone pulse, 70 (79.5%) received IVIG, 37 (42.0%) received cyclosporine, nine (10.2%) received cyclophosphamide, four (4.5%) received mycophenolate mofetil, 22 (25.0%) received hydroxychloroquine, 18 (20.4%) received thalidomide, 9 (10.2%) received tocilizumab, and 1 (1.1%) received infliximab (Table 2). Cyclosporine and thalidomide have been generally used in combination with other medications. At the last follow-up visit, the median dosage of steroids was taped to 0.05 mg/kg. Ten (11.4%) patients reached steroid discontinuation. For patients reaching glucocorticoid discontinuation, six (60.0%) were on tofacitinib monotherapy, one (10.0%) was on methotrexate monotherapy and three (30.0%) were on hydroxychloroquine (HCQ) monotherapy.

**Table 2:** Previous and follow-up medication

Treatment	Treatment before the start of JAKi	Treatment at the start of JAKi	Treatment after the start of JAKi
Glucocorticoid	88 (100%)	81 (92.0%)	781 (88.7%)
Methylprednisolone pulse	51 (57.9%)	18 (20.4%)	1 (1.1%)
Intravenous immunoglobulin	70 (79.5%)	34 (38.6%)	12 (13.6%)
Methotrexate	50 (56.8%)	46 (52.3%)	41 (46.6%)
Cyclosporin	37 (42.0%)	32 (36.4%)	29 (33.0%)
Cyclophosphamide	9 (10.2%)	5 (5.7%)	3 (3.4%)
Mycophenolate mofetil	4 (4.5%)	2 (2.3%)	5 (5.7%)
Thalidomide	18 (20.4%)	14 (15.9%)	12 (13.6%)
Hydroxychloroquine	22 (25.0%)	14 (15.9%)	11 (12.5%)
Tocilizumab	9 (10.2%)	1 (1.1%)	
Infliximab	1 (1.1%)		
Adalimumab			1 (1.1%)

### Tofacitinib efficacy

**Fig. 1:**
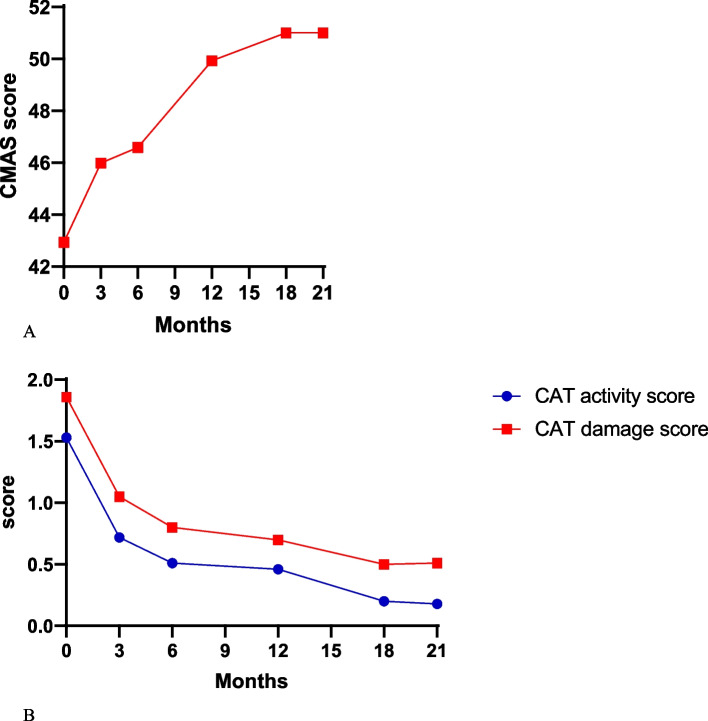
Vital measurement of skin and muscle involvement of JDM at baseline and follow-up visit. **A** CMAS. **B** CAT-BM (CAT activity score and CAT damage score)

Disease activity was improved in patients after at least 3 months of tofacitinib treatment. CAT-BM, CMAS (Fig. 1), and pulmonary function progressed significantly. At the time of tofacitinib introduction (baseline), the median CAT-BM activity score was 1.53 ± 0.15 and decreased significantly during each follow-up visit (3, 6, 12, 18, and 21 months). CAT-BM damage score also dropped significantly (3, 6, 12, 18, and 21 months). CMAS in baseline was 42.94 ± 1.00 and it increased to 51.00 ± 0.40 at 21 months. Significant improvements of CMAS and CAT-BM during each follow-up visit indicated the efficacy of tofacitinib in skin and muscle manifestations. Fifty-three (60.2%) refractory JDM patients presented with pulmonary involvement recovered with no respiratory symptoms and had a reduction in the size or number of lesions under high-resolution computerized tomography (HRCT).

**Fig. 2:**
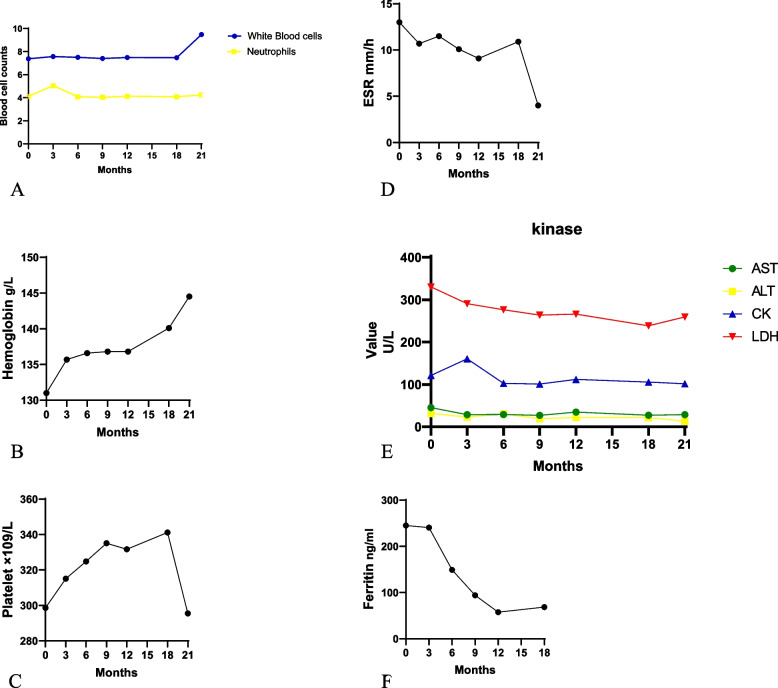
Median value of Blood cells, hemoglobulin, platelet, erythrocyte sedimentation rate, kinases, and ferritin at baseline and follow-up visit. **A** White blood cell. **B** Hemoglobulin. **C** Platelet. **D** Erythrocyte sedimentation rate. **E** Kinases. **F** Ferritin

There was no significant change in the level of white blood cells, neutrophils, hemoglobin, platelet, and erythrocyte sedimentation rate before and after tofacitinib treatment (Fig. 2A–D). In terms ofe kinases, most patients had normal kinases during baseline and follow-up (Fig. 2E). The AST level was 45.4 ± 5.8 U/L at baseline and it decreased significantly at 3, 6, 9, and 12 months. The serum level of ALT decreased significantly during follow-up (3,6,9 and 12 months). CK level at baseline was 121.2 ± 16.2 U/L which was in the normal range. The average LDH level at the tofacitinib baseline was 330.2 ± 16.1 U/L and decreased significantly at 3, 6, 9, and 12 months. Ferritin level at baseline was 245.2 ± 63.2 μg/L, significant decrease in ferritin was found after 3 months of therapy (Fig. 2F). At baseline, 3 patients should be considered the diagnosis as macrophage activation syndrome in accordance with sJIA-MAS criteria [11]. However, they can’t be diagnosed as macrophage activation syndrome since these patients’ temperature fell into a normal range and was not at hyper-inflammatory state.

During disease progression, calcinosis emerged in 3 (3.4%) patients after tofacitinib started. At the last follow-up visit, 15 (75%) patients presented with calcinosis showed a reduction in the size or number of calcinosis, and calcinosis completely resolved in 5 (25%) patients.

**Fig. 3:**
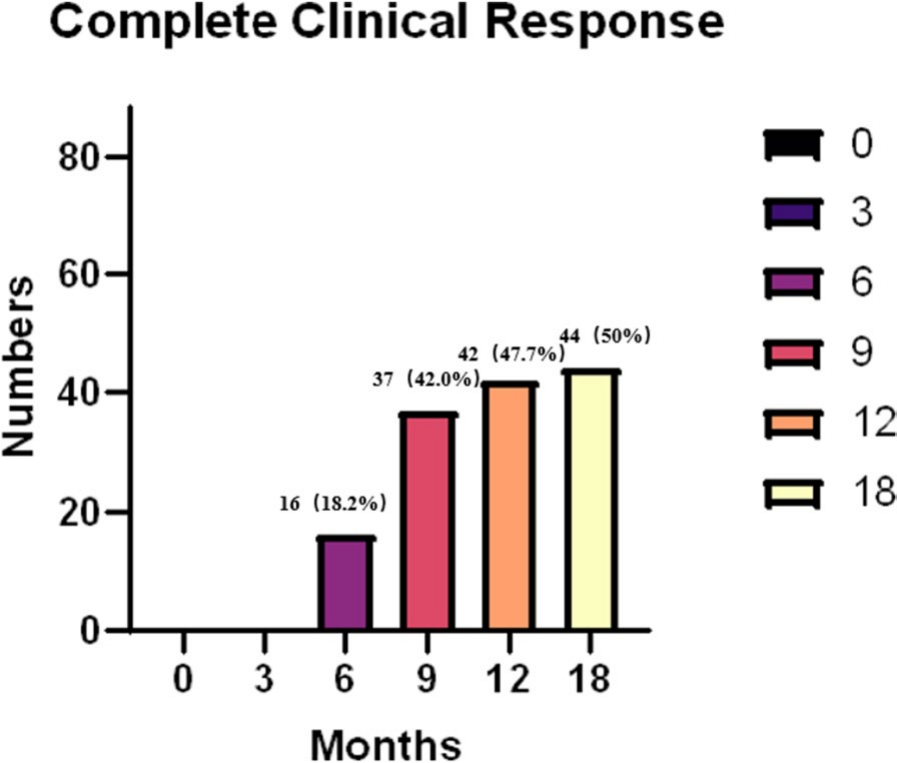
Complete clinical response rate. The number of patients achieved complete clinical response after JAKi at the follow-up visit. Sixteen patients reached complete clinical response at 6 months. Thirty-seven patients reached response in 9 months, 42 patients in 12 months, and 44 patients in 18 months. In total, 44 refractory patients reached a response

Sixteen (18.2%) patients reached IMACS-defined complete clinical response after 6 months of therapy. Thirty-seven (42.0%) patients reached complete clinical response in 9 months, 42 (47.7%) patients reached complete clinical response in 12 months and 44 patients reached complete clinical response in 18 months. In general, 44 (50.0%) refractory patients reached complete clinical response (Fig. 3). Of patients who reached clinical response, 2 (2.3%) of them had medicine escalation. One got medicine escalation one month after reaching a complete clinical response and reached a response again 8 months later. One got medical escalation 3 months after reaching a complete clinical response and now remains in an active state. Most patients reached complete clinical response at 9 months since active skin rashes and muscle weakness served as the indicator of tofacitinib start. No patient in this study reached complete clinical remission.

Among patients carrying positive MSAs and MAAs, 9 (19.1%) of them reached complete clinical response at 6 months, 19 (40.4%) patients reached complete clinical response in 9 months, 22 (46.8%) reached complete clinical response in 12 months and 26 (55.3%) reached complete clinical response in 18 months. The total response rate was 55.3%. Thirteen out of nineteen (68.4%) patients with anti-MDA5 antibodies and 8 (66.7%) patients with anti-NXP2 antibodies reached complete clinical response. No difference was reported between patients carrying MSAs and negative MSAs after finishing the chi-square test (*P* = 0.801, 0.742, 0.853, and 0.285 in 6, 9, 12, and 18 months).

### Adverse effect

Common adverse effects (e.g., tuberculosis, perforation of the gastrointestinal system, thrombosis, and allergic reactions) were not reported in our study. Only one patient had herpes zoster infection in 9 months after initiation. After drug withdrawal, herpes recovered and tofacitinib was given again.

## Discussion

Juvenile dermatomyositis is a multi-system-affected inflammatory myopathy. Skin and muscular involvements were prominent, and other systems were also affected [12]. Despite combined prednisolone and methotrexate being recommended as first-line therapy and beneficial, nearly 40–60% of patients are still in active state [6]. The prognosis and long-term outcome of JDM were poor due to its chronic features and overall high mortality rate. Hence, early diagnosis and treatment are essential and new immunosuppressants and biologics agents are required for the treatment of refractory JDM. It is reported that increased IFN, IFN-regulated genes, and IFN-regulated proteins were found in the skin and muscle of JDM patients [7]. As revealed by in vitro studies, activation of the IFN pathway can mimic dermatomyositis (DM) pathological findings like muscle atrophy and vasculopathy. After using ruxolitinib (another first-generation JAK inhibitors) in vitro, the above-mentioned DM-like findings could be inhibited. Later, through treating 4 adult DM patients with ruxolitinib, the expression levels of IFN-related genes were lower and skin disease activity improved [13]. Tofacitinib is a first-generation JAK inhibitor that was approved in China since 2017. Sabbagh et al. reported 2 cases of anti-MDA5 antibody-positive juvenile DM with refractory skin, muscle, and pulmonary involvement, as well as elevated IFN score. Methylprednisolone pulse, IVIG, and mycophenolate mofetil showed no help with disease flares implying their refractoriness. After several months of tofacitinib treatment, their physician’s global assessment (PGA), manual muscle testing (MMT8), and cutaneous disease area and severity index (CDASI) scores decreased significantly and achieved moderate improvements in disease activity [14]. Antecedent researchers uncovered the possibility of blockage of the IFN pathway through JAK inhibitors in treating refractory JDM patients.

Our study was a retrospective study of refractory JDM patients from different centers in China. After assessing 88 refractory JDM patient’s clinical features, laboratory tests, diagnostic imaging tests, treatment options, and follow-up every 3 months, we can evaluate tofacitinib efficacy through this study.

Eight-eight refractory JDM patients were included, and a female predominance was reported. Skin and muscle were the most affected system. Median age of disease onset was 82.0 ± 4.1 months. In accordance with CAT-BM and CMAS scores, most patients were classified into mild and moderate states at first description. As indicated by the result of HRCT, 50 patients were subjected to interstitial lung disease. Severe myositis, dysphagia, cardiopulmonary involvement, and poor response to steroid therapy were correlated with poor prognosis [15]. Taken together with the failure of traditional combined and biologic therapy, most patients included in this study at first description were severe and correlated with poor prognosis. The average time of tofacitinib prescription was 22.5 ± 25.4 months, the differences between the time of first description and started using tofacitinib therapy primarily arose from different times of first description. The longest time of starting tofacitinib from the first description was 141 months with disease onset in 2010. In accordance with patients who had positive MSAs and MAAs, the NXP2 and MDA5 were the most abundant and the complete clinical response rates between different MSAs were nearly identical. Another center in China using tocilizumab in refractory MDA5 + RP-ILD patients yielded promising results regarding symptoms and HRCT findings [16]. Nine patients who tried tocilizumab in our study showed improved HRCT findings, whereas none of them reached complete clinical response. After at least 3 months of tofacitinib treatment, patients were improved in multiple aspects. Skin lesions were examined through CAT-BM, muscle strength through CMAS and kinases, and pulmonary function through HRCT. The prominent enhancement was shown, indicating that tofacitinib can be conducive to skin, muscle, and pulmonary manifestations. Serum levels of blood cells and ESR were unremarkable during a follow-up visit. Most patients had normal serum levels of kinases at baseline and follow-up visits.

Previous studies have found that serum ferritin > 1000 mg/ml served as a negative prognostic factor of anti-MDA5 + dermatomyositis [17]. A significant decrease of ferritin was identified at 3 months correlated with existing studies. Three patients considered the diagnosis as MAS in accordance with sJIA-MAS criteria. Central nervous system dysfunction, hemorrhage, and hepatomegaly are vital clinical manifestations of MAS. These 3 patients were not in a hyper-inflammatory state. Increased levels of AST and decreased fibrinogen were due to liver dysfunction.

The initial prednisolone dose was 2 mg/kg and it gradually taped to the median level of 0.05 mg/kg. For 10 patients with glucocorticoid discontinuation, six were on tofacitinib monotherapy, one was on methotrexate monotherapy and three were on HCQ monotherapy in a relatively low dose suggesting that tofacitinib may be useful as monotherapy.

Calcinosis is hard to treat and it is correlated with a high morbidity rate. Hypothesis has been proposed that the pathogenesis could be excessive calcium storage in mitochondria and release through the STAT 3 pathway [18]. Thus, the tofacitinib blocking STAT3 pathway can serve as a therapeutic approach for calcinosis. Seventeen (19.3%) patients had calcification at first description. With disease progression, calcinosis emerged in 3 (3.4%) patients, so at the time of tofacitinib initiation. Thus, a total of 20 (22.7%) patients have calcinosis at the time of tofacitinib initiation. Size or number reduction was seen in 15 (75%) patients, and the completely resolved calcinosis was identified in five patients. Nevertheless, drugs cannot be stopped immediately after calcinosis was resolved.

The total complete clinical response rate reached 50% and most patients met complete clinical response in 9 months after prescription. Different MSAs showed similar response rates despite different outcomes which coordinated with a retrospective study with 10 patients. Five out of 10 patients reached CID despite being subjected to a wide variety of clinical manifestations and autoantibodies [10]. In terms of safety, only one patient had herpes zoster infection and recovered after the drug stopped which was tolerated.

Our study is unique because it has been the largest research to evaluate the efficacy of tofacitinib in the world of refractory JDM patients. The sample size was increased and the time of follow-up was extended as compared with than before. But several limitations existed in our study. Since the Pediatric Rheumatology International Trials Organization (PRINTO) defines clinically inactive disease (CID) using CK, CMAS, MMT, and visual analog scale (VAS) [19], considerable studies used CID to evaluate the efficacy of treatments. Since some clinical data were lacking, patients cannot be assessed in accordance with PRINTO CID criteria. The role of tofacitinib in the IFN pathway cannot be expressed due to the lack of data on IFN, IFN-associated gene expression, and the cytokine level in baseline and follow-up visits. In-depth research should place a focus on the correlation of JAK inhibitors with the IFN pathway, IFN-associated gene, and gene expression. Lack of carbon monoxide diffusion capacity (DLCO) results and the analysis only based on HRCT.

As mentioned above, JAK inhibitors-tofacitinib treatment had yielded complete clinical response and improvement in several refractory JDM patients without severe adverse reactions. Tofacitinib can serve as a therapeutic option for JDM patients. Subsequent prospective studies should be conducted to primarily investigate the mechanism of tofacitinib in the IFN pathway. Besides, clinical trials should be performed through efficacy between tofacitinib and conventional treatment strategy in refractory JDM patients.

## Conclusion

After initiating tofacitinib, skin and muscle disease reductions were found. Tofacitinib was well tolerated without any severe adverse effects.

## Data Availability

Condensed anonymized data are available from the corresponding author on reasonable request.

## Abbreviations

JDM: Juvenile dermatomyositis
JAK: Janus kinase
JAKi: JAK inhibitors
IIM: Idiopathic inflammatory myopathies
MSAs: Myositis-specific antibodies
MAAs: Myositis-associated antibodies
RP-ILD: Rapidly progressive interstitial lung disease
MAS: Macrophage activation syndrome
MTX: Methotrexate
IVIG: Intravenous immunoglobulin
MMF: Mycophenolate mofetil
CSA: Cyclosporin A
IFN: Interferon
IRG: Interferon-regulated gene
STAT: Signal Transducers and Activators of Transcription
CID: Clinical inactive disease
ELISA: Enzyme-linked immunosorbent assay
CAT-BM: Cutaneous Assessment Tool-binary method
CMAS: Childhood Myositis Assessment Scale
AST: Aspartate aminotransferase
ALT: Alanine aminotransferase
CK: Creatine kinase
LDH: Lactate dehydrogenase
IMACS: International Myositis Assessment and Clinical Studies Group
OI: Opportunistic infections
HCQ: Hydroxychloroquine
PRINTO: Pediatric Rheumatology International Trials Organization
PGA: Physician’s global assessment
MMT8: Manual muscle testing
CDASI: Cutaneous disease area and severity index
ILD: Interstitial lung disease
VAS: Visual analog scale
DLCO: Carbon monoxide diffusion capacity
